# Comparison of Controllability Features of Extractive and Pressure Swing Distillations on the Example of Tetrahydrofuran Dewatering

**DOI:** 10.1021/acsomega.1c04606

**Published:** 2021-12-15

**Authors:** Jonathan Wavomba Mtogo, Andras J. Toth, Agnes Szanyi, Péter Mizsey

**Affiliations:** † Department of Chemical and Environmental Process Engineering, Budapest University of Technology and Economics, 1111 Budapest, Hungary; ‡ Department of Fine Chemicals and Environmental Technology, University of Miskolc, 3515 Miskolc, Hungary; § Chemical Engineering Division, Kenya Industrial Research and Development Institute, P.O. Box 30650, 00100 Nairobi, Kenya

## Abstract

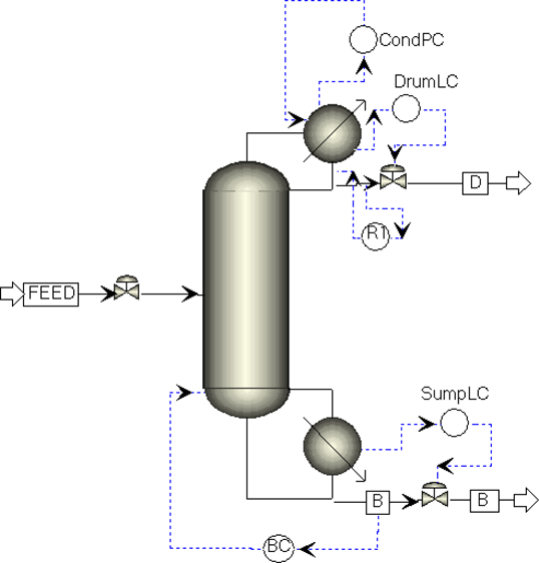

The controllability study is an integral part of chemical process design. In this work, the controllability of two special distillation techniques, extractive distillation and pressure swing distillation, designed for the separation of azeotropic mixtures is investigated with dynamic tools. The control design interface of Aspen Plus and Matlab are applied for the modeling and evaluation of the two systems. Dynamic controllability indices are determined and aggregated in a desirability function. The results are compared to obtain efficient help for process design activity. The pressure swing distillation shows significantly better controllability features than the extractive distillation. The reason can be the fact that in the case of the extractive distillation, a third compound, the extractive agent, is added to the system to carry out the separation, therefore making the system more complex. As far as the selection of manipulated variables is concerned, in the case of the extractive distillation, the reflux flows should be preferred to the reflux ratios but in the case of the pressure swing distillation, the reboiler heat loads are preferred to the reflux ratios since those are closer to the controlled compositions. Both separation systems show worse controllability features if the product purity requirement is approaching to the pure products, that is, close to 100%. Although the energy consumption of the pressure swing distillation is higher than that of the extractive distillation, it has the inherent feature that it can be automatically heat integrated due to a column operated at high pressure and, as a consequence, higher temperatures.

## 1 Introduction

Tetrahydrofuran (THF) is an excellent solvent and it is commonly utilized within the chemical industry. It is also often used as a starting material or chemical intermediate in the synthesis of various products such as adhesives, coating products, and cleaning products. The water miscibility of THF and its high vapor pressure promotes its transfer to the atmosphere and to both surface and ground waters.^1^ THF in the environment affects the health of both humans and animals. It affects the central nervous system and liver and has the potential to induce cancer.^2^ For this reason, THF should be controlled during production from getting into process effluents.

THF is produced in most commercial processes as a mixture with water. Ordinary distillation cannot separate the THF–water mixture because of the formation of a minimum boiling homogeneous azeotrope. Several alternative distillation techniques can be considered for azeotropic mixture separation. Among them, extractive distillation (ED)^3^ and pressure swing distillation (PSD)^4^ are two techniques that have been extensively used in the commercialized separation of the mixture of THF–water. The extractive distillation (ED) process requires an additional agent in the form of an entrainer or a solvent. From the literature, various design criteria guiding the selection of a suitable entrainer such as capacity, stability, noncorrosivity, cost, volatility, and selectivity are highlighted.^5−7^ The pressure swing distillation (PSD) process, on the other hand, does not involve any additional entrainer or solvent. As a result, it is considered to be an eco-friendly process.

Extractive distillation is the most widely used method for THF–water separation, and several steady state designs are available. Xu and Wang^3^ studied the separation by extractive distillation and demonstrated the design option for the choice of entrainer. An experimental work has also been used to compare various entrainers for THF–water separation.^7^ For the separation of an equimolar THF–water mixture, Ghuge et al.^8^ simulated both extractive distillation and pressure swing distillation. Their works have been on selection of the suitable process on the basis of the total annual cost (TAC). They have obtained the conclusion that ED has a lower TAC compared to the PSD. These studies are based on steady-state process design.

However, dynamic controllability studies have to be carried out to validate the applicability of the technology in the industry.^9−11^ Such studies can be carried out through control structure design, which is a well-known field.^12,13^ Douglas^14^ has described the conceptual design of chemical processes. Ziegler and Nichols^15^ have developed the principle of process controllability, which is a process of achieving and maintaining desirable equilibrium values. Other authors, for example, Emtir and Fonyo,^16^ have documented that the mutual influence of process design and control may complicate the process design activity. It is necessary that the synthesis of control structures should be considered over the early stage of the process design in order to make a complete assessment of the design alternatives.^17^

The control structure design studies should be performed during the process operation analysis in the presence of disturbance. The rate of feed flow and its composition always meet disturbance in the distillation processes.^18^ A good technology ought to have the capability to cope with the disturbance to a certain extent. Based on their steady-state findings, various researchers have investigated the dynamic control of EDs and PSDs.^19−21^ Luyben’s work detailed the dynamic control of the PSD with heat integration for separating a THF–water azeotrope.^22^ These researchers have presented control structures and tuning procedures for use in Aspen Plus Dynamics for the processes. Iqbal et al.^21^ presented the control structure of ED using a Hi-Selector control logic to maintain the total entrainer flow rate.

In this work, for the ED and PSD processes, we investigate the dynamics and stability by designing control strategies to effectively handle the feed rate and composition disturbances with a focus on maintaining product purity. We study the interactions among the control loops as well as how the pairings of controlled and manipulated variables affect the behavior of the separation systems at different purity levels. The control design interface (CDI) feature of Aspen Plus Dynamics is used to accomplish linearization and controllability indices in the frequency domain. Controllability indices are calculated using Matlab and then aggregated in one parameter with the use of the desirability function. These controllability indices are the Morari resiliency index (MRI), conditioning number (CN), and the relative gain array number (RGAno). MRI for the open-loop transfer function is described as the minimal singular value for a specific input and output direction. The highest is the MRI, and the best is the system. The conditioning number is the maximum over the minimum singular value of the process open loop frequency function. By using MRI and its associated CN, an open loop system can be evaluated to withstand model uncertainties and disturbances.^23^ The relative gain array number (RGAno, relative gain number minus 1) is used to find the best manipulated and controlled variable pairing. The lower the relative gain array number, the lower the system interaction at that frequency, with zero being ideal.^12^

## 2 Steady-State Designs

### 2.1 Extractive Distillation

This simulation is on the basis of a case study of an equimolar feed of THF–water mixture. The feed flow rate is 100 kmol/h. DMSO is selected as the entrainer. Deorukhkar et al.^24^ and Ghuge et al.^8^ have explored the suitability of DMSO and reported that it is efficient for the separation. The most suitable thermodynamic property model for this process is NRTL.^6,25^ The binary interaction parameters for the components were regressed by Aspen Plus and are shown in Table 1.

**Table 1:** NRTL Binary Interaction Parameters

component *i*	component *j*	*A_ij_*	*A_ji_*	*B_ij_*	*B_ji_*	α_*ij*_
THF	water	1.274	4.919	157.781	–733.402	0.473
THF	DMSO	3.8117 × 10^–5^	–7.6568 × 10^–6^	347.549	73.937	0.300
water	DMSO	–1.2449	1.7524	586.801	–1130.215	0.300

**Figure 1:**
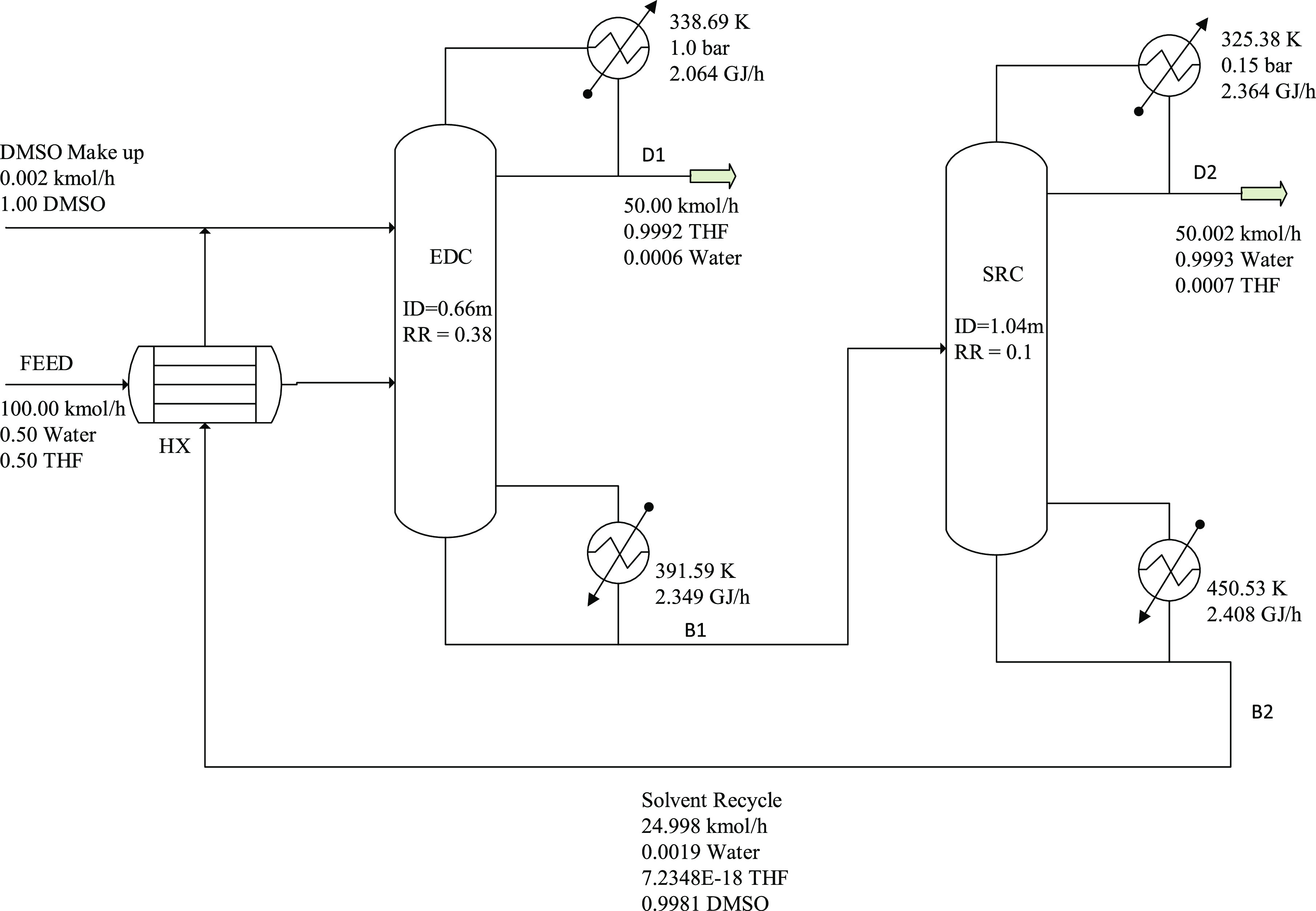
Flowsheet of extractive distillation.

The various design variables for the two columns are optimized with the use of the Aspen Plus built-in sensitivity analysis functionality. The sensitivity analysis is done according to the method described by Ghuge et al.^8^ The molar reflux ratio, total number of theoretical stages for the extractive and the solvent recovery columns (EDC and SRC), the mixtures of the feed stage and the solvent feed stage for the extractive column, and the feed stage location for the solvent recovery column are considered. The work is completed for both 95.0 and 99.9 mol % THF product purities. Figure 1 shows the flowsheet of the extractive distillation process as drawn by Aspen Plus.

Table 2 shows the design parameters of the distillation columns.

**Table 2:** Design Parameters for the THF–Water Columns in Extractive Distillation

design parameters	EDC	SRC
molar reflux ratio	0.38	0.1
number of theoretical stages	21	14
entrainer feed stage	5	
feed stage	17	6
entrainer feed rate (kmol/h)	25	

### 2.2 Pressure Swing Distillation

**Figure 2:**
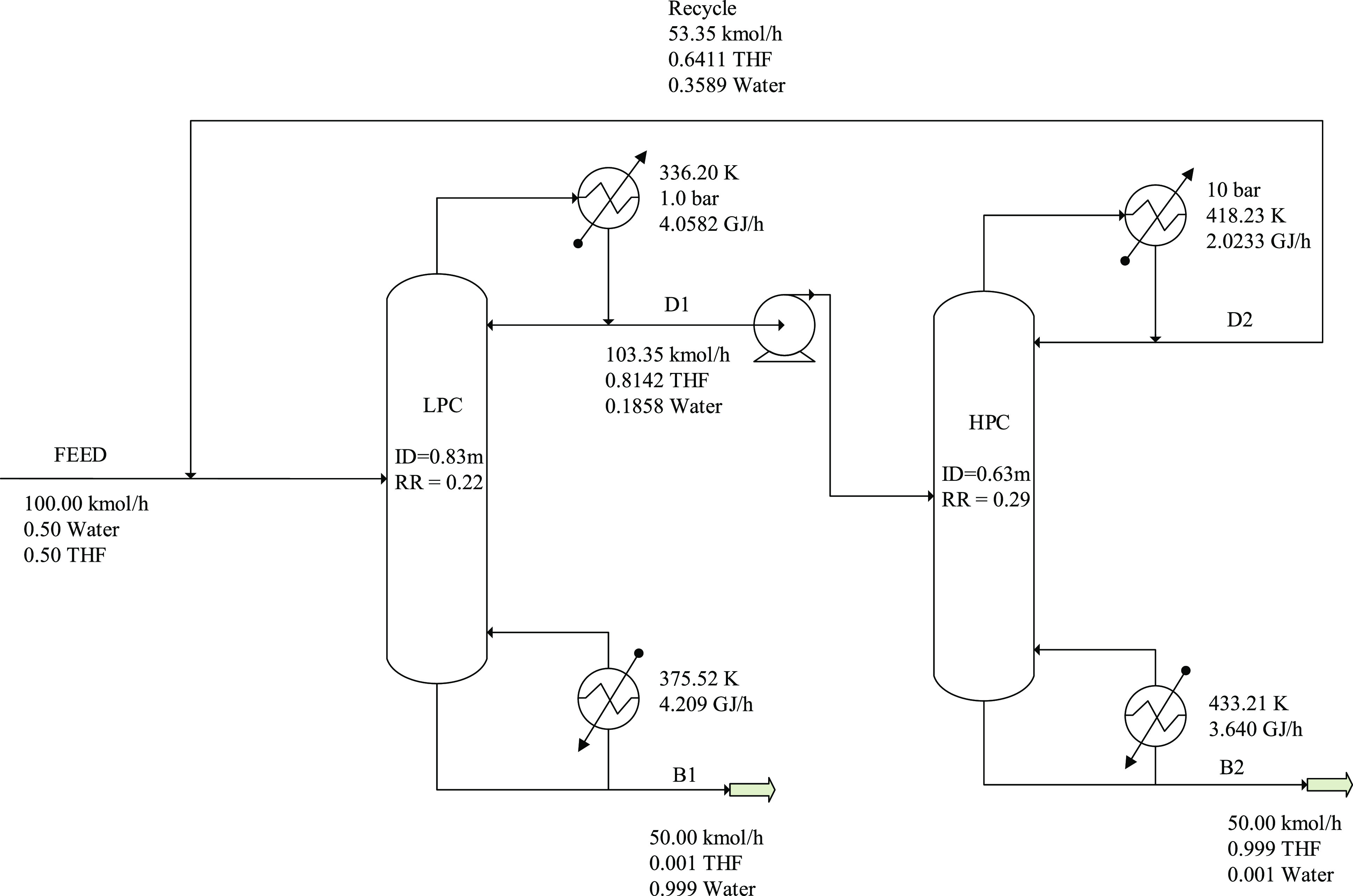
Flowsheet for pressure swing distillation.

The pressure swing distillation flowsheet is shown in Figure 2. The binary feed condition is set equivalent to that of the extractive distillation. The two distillation columns are operated at different pressure levels to take advantage of the pressure sensitivity of the THF–water azeotrope. The first column (low-pressure column, LPC) is operated at 1 bar, whereas the second column (high-pressure column, HPC) is operated at 10 bar. The basis for the selection of the operating pressure for the columns was the effect of pressure on the azeotropic composition and on the reboiler temperatures. The variation of the composition of the azeotrope with pressure is shown in Table 3. When there is a large pressure difference, there is a corresponding shift in the azeotropic composition, resulting in low recycle flows and energy consumption. In the LPC, pressures less than 1 bar would necessitate the use of costlier chilled water in the condenser and therefore this option is avoided. As a result, in the LPC, 1 bar is selected. The operating pressure for the HPC is specified so that high-pressure steam can be still used as the utility in the reboiler. Moreover, there is only minimal change in the azeotropic composition above 10 bar. Therefore, a pressure of 10 bar is selected in the HPC.

**Table 3:** Pressure Change Effect on Azeotropic Composition of the THF–Water System

pressure (bar)	azeotropic composition (mole fraction of THF)	boiling temperature of azeotrope (K)
1	0.8287	336.55
2	0.7775	357.65
3	0.7442	371.35
4	0.7190	381.65
5	0.6980	390.15
6	0.6814	397.25
7	0.6666	403.55
8	0.6535	409.15
9	0.6418	414.15
10	0.6312	418.85
11	0.6215	423.05

Other design parameters are shown in Table 4.

**Table 4:** Design Parameters for the THF–Water Columns in Pressure Swing Distillation

design parameters	LPC	HPC
mole reflux ratio	0.22	0.29
number of theoretical stages	13	16
feed stage	10	8
pressure (bar)	1	10

### 2.3 Energy Consumptions

Based on the steady-state simulations, the energy requirements of the two separation alternatives for both product purities are determined and presented in Table 5*.*

**Table 5:** Heating Energy Requirements of the Alternatives Studied

	Extractive distillation		Pressure swing distillation	
purity mol %	95 mol %	99.9 mol %	95 mol %	99.9 mol %
Heating energy GJ/h	4.55 GJ/h	4.76 GJ/h	6.08 GJ/h	7.85 GJ/h

The energy requirement results are in agreement with those of Luyben.^19^ In spite the fact that the pressure swing distillation system needs more energy than the extractive distillation system, it has the advantage that it can be automatically energy integrated due to the high-pressure column. This column, as a consequence of the higher pressure, operates on higher temperatures.

The amount of energy required increases with the purity of the product. This is also consistent with other literature.^8^

## 3 Controllability Analysis

The reflux drum sizes and the column sump sizes are calculated before the simulation is exported into Aspen Dynamics. A 5 min hold up if 50% filled heuristic is used.^10^ The ratio of height to diameter is set as 2. To allow for dynamic operation, pumps and valve pressures are set. As suggested from the literature, PI controllers are used.^19,21^ Variables are chosen to manipulate the product compositions based on the control structure design heuristic criterion that the nearest potential manipulated variable is chosen. The first manipulated variable in each pairing determines the product A composition, whereas the second determines the product B composition. For the extractive distillation, one extra control loop, that is, pairing is needed for the control of the purity of the entrainer. The pairings are shown in Table 6.

**Table 6:** Pairing of the Control and Manipulated Variables

separation system	extractive distillation	pressure swing distillation
controlled compositions	X_T_-X_W_-X_S_	X_W_-X_T_
set 1 of manipulated variables	R1-R2-Q2	R1-R2
set 2 of manipulated variables	L1-L2-Q2	Q1-Q2

The built-in Ziegler–Nichols tuning method of ASPEN is used. Time constants are determined on the basis of load rejection investigations with disturbances in the feed composition and feed flow rate.^26^ The methodology for controllability analysis is established on the basis of the study by Gabor and Mizsey,^17^ which is the fastest methodology for calculating the controllability indices within the frequency domain.

In this method, the state space representation for the dynamic system is obtained through the CDI module of Aspen Plus Dynamics. A script that matches the input and output variables is created, and the desired matrices are calculated. In order to run the script, the input variables selected must be fixed, whereas the output variables must be free. If the simulation is run until a steady state is reached, then the state space matrices are generated by CDI. On the basis of the matrices, the controllability indices can be calculated in the function of the frequency. A Matlab code is written that presents graphs of the controllability indices as functions of frequency.

These controllability indices include the following:

- Morari resiliency index (MRI),
- condition number (CN), and
- relative gain array number (RGAno).

MRI is defined as the least singular value of the open loop frequency function matrix for the process.^12^ A large MRI value indicates better controllability.

The CN is defined as the ratio of the highest and lowest singular values of the process open loop frequency function matrix. The matrix is considered to be ill-conditioned if it exceeds 100, and therefore the process is less controllable. The generally acceptable range of CN value is 1–10.

In the case of RGAno, there exists a square matrix (RGA) for a nonsingular square matrix *G*. The definition of RGA is as follows:
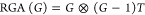
where ⊗ indicates the multiplication of element by element. *T* indicates the transponation of the corresponding matrix. The RGA value denotes the interactions among the control loops in the process. RGAno is described as follows:
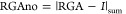
where *I* is the unit matrix.

Pairings with weaker interactions are preferred. These are depicted by low RGAno.

It is preferred that the three controllability indices are aggregated in one number. The so-called desirability function^27,28^ is selected for such an aim. Individual desirability functions are calculated for each controllability index, and their geometric average is calculated to form the desirability function for a system using the formulas below:
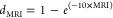

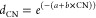

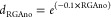

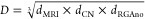
where *a* and *b* are 0.0004 and 0.007, respectively, and *D* is the aggregated desirability function for a system.

This aggregated value represents a way to perform direct comparisons between different alternatives, that is, controllability alternatives. Desirability values closer to 1 are preferable as they indicate good process controllability.

## 4 Results and Discussion

**Figure 3:**
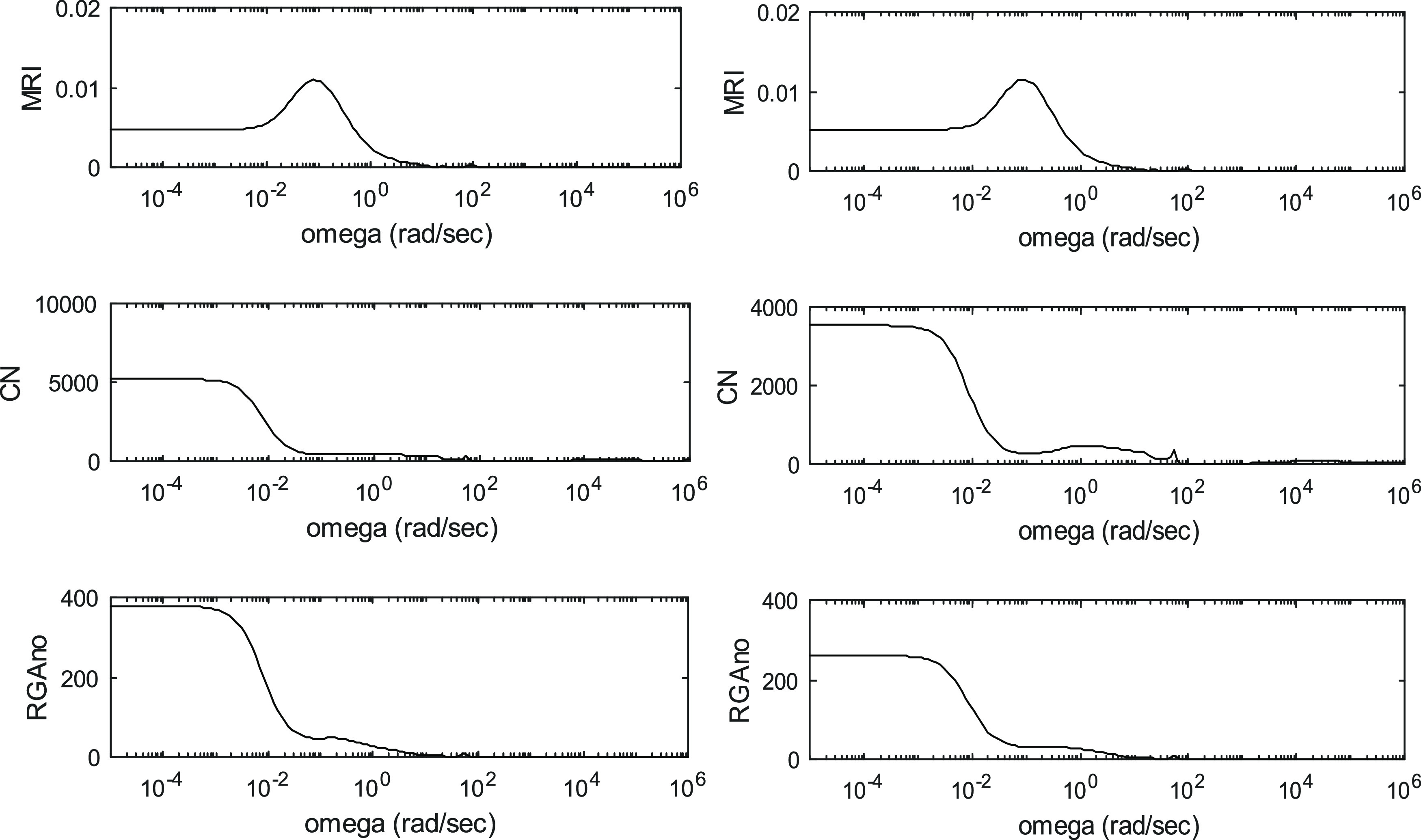
Controllability indices of ED in the case of 95 mol % product purity for the R1-R2-Q2 and L1-L2-Q2 manipulated variable sets.

**Figure 4:**
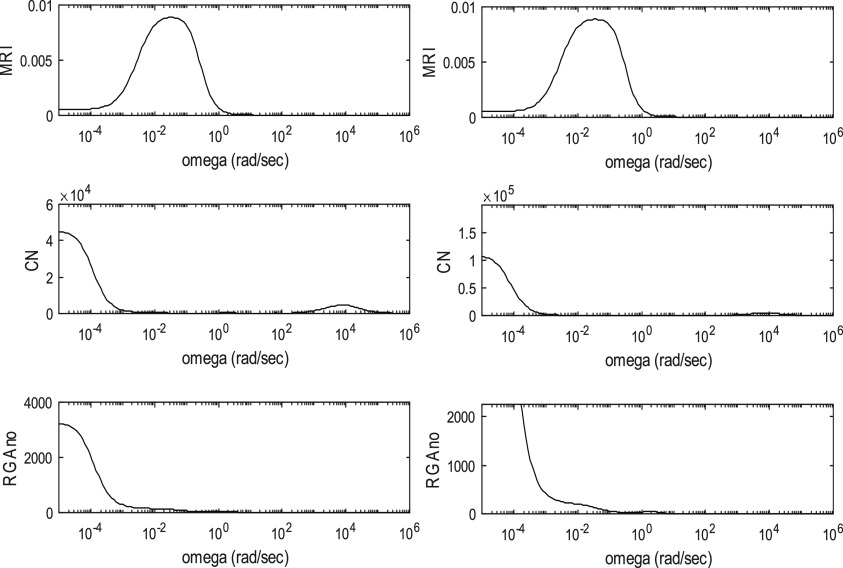
Controllability indices of ED in the case of 99.9 mol % product purity for the R1-R2-Q2 and L1-L2-Q2 manipulated variable sets.

**Figure 5:**
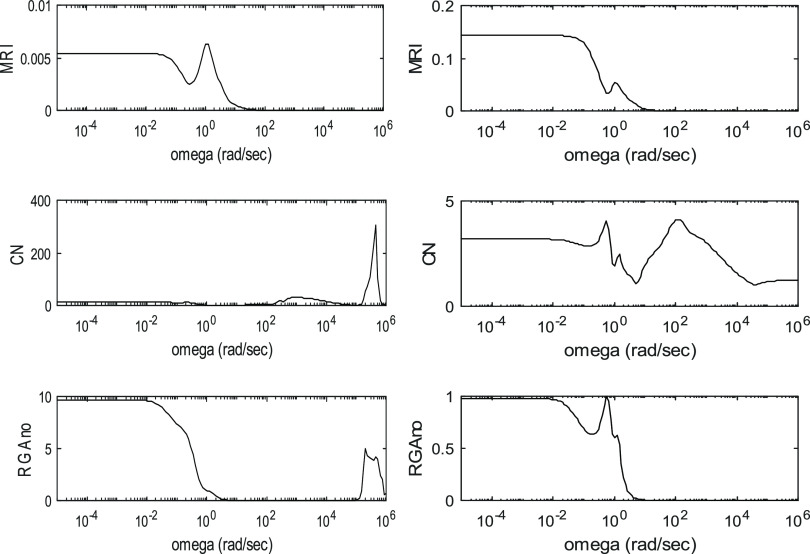
Controllability indices of PSD in the case of 95 mol % product purity for the R1-R2 and Q1-Q2 manipulated variable sets.

**Figure 6:**
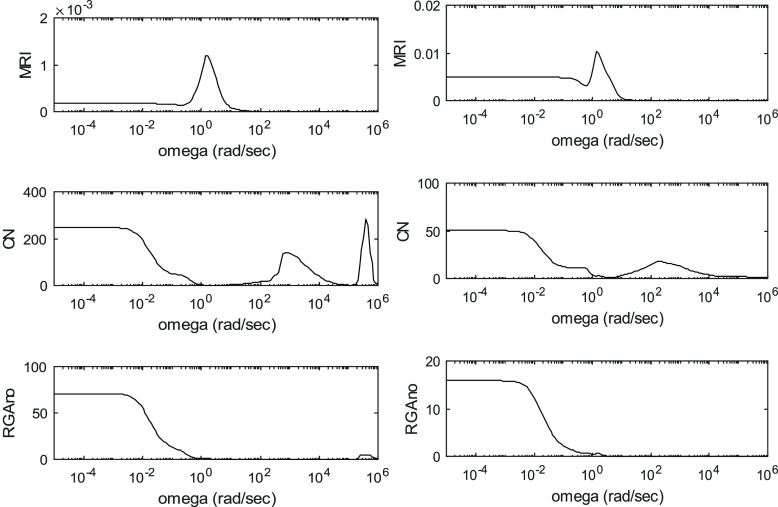
Controllability indices of PSD in the case of 99.9 mol % product purity for the R1-R2 and Q1-Q2 manipulated variable sets.

The controllability indices for the different processes are shown in Figures 3–6. These are depicted as functions of angular frequency.

Controllability indices and desirability values for the systems investigated are presented in Table 7. The heating requirements are also indicated there. The angular frequency is computed from the time constant, which is derived from load rejection analysis of the individual systems.

**Table 7:** Controllability Indices and Desirability and Values for the Systems

	extractive distillation				pressure swing distillation			
purity of THF	95 mol %		99.9 mol %		95 mol %		99.9 mol %	
control structure	R1-R2-Q2	L1-L2-Q2	R1-R2-Q2	L1-L2-Q2	R1-R2	Q1-Q2	R1-R2	Q1-Q2
time constant (h)	0.41 h	0.41 h	0.45 h	0.45 h	0.13 h	0.13 h	0.3 h	0.3 h
frequency (rad/s)	6.68 × 10^–4^	6.68 × 10^–4^	6.68 × 10^–4^	6.68 × 10^–4^	2.14 × 10^–3^	2.14 × 10^–3^	9.26 × 10^–4^	9.26 × 10^–4^
MRI	4.73 × 10^–3^	5.17 × 10^–3^	1.44 × 10^–3^	1.41 × 10^–3^	5.44 × 10^–3^	1.44 × 10^–1^	1.72 × 10^–4^	5.01 × 10^–3^
CN	5.17 × 10^3^	3.51 × 10^3^	5.00 × 10^3^	3.42 × 10^3^	1.43 × 10^1^	3.19 × 10^0^	2.47 × 10^2^	5.01 × 10^1^
RGAno	3.74 × 10^2^	2.59 × 10^2^	6.01 × 10^2^	4.13 × 10^2^	9.69 × 10^0^	9.81 × 10^–1^	7.10 × 10^1^	1.59 × 10^1^
*d*_MRI_	4.62 × 10^–2^	5.04 × 10^–2^	1.43 × 10^–2^	1.40 × 10^–2^	5.30 × 10^–2^	7.62 × 10^–1^	1.72 × 10^–3^	4.89 × 10^2^
*d*_CN_	1.96 × 10^–16^	2.19 × 10^–11^	6.21 × 10^–16^	4.09 × 10^–11^	9.04 × 10^–1^	9.78 × 10^–1^	1.78 × 10^–1^	7.04 × 10^–1^
*d*_RGAno_	5.50 × 10^–17^	5.70 × 10^–12^	8.08 × 10^–27^	1.13 × 10^–18^	3.79 × 10^–1^	9.07 × 10^–1^	8.26 × 10^–4^	2.05 × 10^–1^
aggregated desirability	7.92 × 10^–12^	1.85 × 10^–8^	4.16 × 10^–15^	8.66 × 10^–11^	2.63 × 10^–1^	8.77 × 10^–1^	6.32 × 10^–3^	1.92 × 10^–1^

The comparison of the desirability indices is clear:

- The pressure swing distillation has significantly higher values than the extractive distillation system, indicating better controllability features;
- Both separation systems can be definitely better controlled at lower purity products, 95 mol %, than in the case of high purities, 99.9 mol %. This is indicated by the desirability indices that are higher with two orders of magnitude than those of the 99.9 mol % product purity case;
- Comparing the two control structures for the extractive distillation, R1-R2-Q2 and/or L1-L2-Q2, that one shows better controllability feature, that is, higher desirability values, where the reflux flows are used as manipulated variables both in the case of extractive distillation and pressure swing distillation;
- In the case of the pressure swing distillation, there are only two control loops and therefore two manipulated variables for the two product compositions. If the heat loads, that is, Q1-Q2 are applied as manipulated variables, then the control shows better controllability features and higher desirability values. This is in agreement with the heuristic, that is, the manipulated variable should be close to the controlled variable.

## 5 Conclusions

Two frequently studied and applied special distillation structures designed for the separation of azeotropic mixtures, the extractive distillation and the pressure swing distillation, are studied from controllability point of view.

According to the results, the pressure swing distillation has significantly better controllability features than the extractive distillation. It can be due to the fact that in the case of the extractive distillation, a third compound, the extractive agent, is added to the separation, making the system more complicated. Also, the time constants of the system determined from step responses are higher in the case of extractive distillation.

Although the pressure swing distillation has higher energy consumption than the extractive distillation, but due to the high-pressure column, there is an inherent possibility for heat integration between the two columns, reducing the energy consumption significantly. It is important to note that heat integration is also possible in extractive distillation but with lower percentage saving.

When the product purity is higher, the systems have higher time constants, and the controllability features become poor. This conclusion is in agreement with the experiences.

As far as the set of the manipulated variables is concerned, the selection of the reflux flow rates as manipulated variables proves to be better over the reflux ratio in the cases of extractive distillation. In the case of the pressure swing distillation, the reboiler heat loads are a better set of manipulated variables. The results are in agreement with the heuristic recommendations, that is,

- if the reflux flowrate is low, then the control of the product composition is usually better with use of reflux flow as the manipulated variable and
- the reboiler heat loads are closer to the controlled variables, therefore these are the better manipulated variables for bottom product composition control.

The results can support the process design step, and the controllability features show preferences to the pressure swing distillation over the extractive distillation.

## Nomenclature

CDI: control design interface
CN: condition number
DMSO: dimethyl sulfoxide
ED: extractive distillation
L: reflux flow
MRI: Morari resiliency index
PSD: pressure swing distillation
R: reflux ratio
RGAno: relative gain array number
THF: tetrahydrofuran
X: composition
